# Draft Genome Sequence of *Thermotoga maritima* A7A Reconstructed from Metagenomic Sequencing Analysis of a Hydrocarbon Reservoir in the Bass Strait, Australia

**DOI:** 10.1128/genomeA.00688-13

**Published:** 2013-09-05

**Authors:** Brodie Sutcliffe, David J. Midgley, Carly P. Rosewarne, Paul Greenfield, Dongmei Li

**Affiliations:** CSIRO Animal, Food and Health Sciences, North Ryde, NSW, Australiaa; CSIRO Mathematics, Informatics and Statistics, North Ryde, NSW, Australiab

## Abstract

The draft genome sequence of *Thermotoga maritima* A7A was obtained from a metagenomic assembly obtained from a high-temperature hydrocarbon reservoir in the Gippsland Basin, Australia. The organism is predicted to be a motile anaerobe with an array of catabolic enzymes for the degradation of numerous carbohydrates.

## GENOME ANNOUNCEMENT

The quality and efficiency of oil recovery from high-temperature oil reservoirs are affected by the activity of endogenous microbes (1). In a step toward unraveling the microbial communities in oil-associated subsurface environments, we examined the metagenome of a hydrocarbon reservoir (named “Tuna”; 38°10′S, 148°25′E) obtained from the A7A well in Gippsland Basin, Australia. The A7A well is ~1,960 m deep and has a temperature of 102°C, a pH of 7.2, and a salinity of 2.68%.

The metagenome was sequenced on the Illumina HiSeq 2000 system, which generated 2 × 100-bp paired-end reads. The resulting ~50 million metagenomic reads were corrected using Blue (http://www.bioinformatics.csiro.au/blue), prior to being assembled using Velvet v1.2.07 (*k* = 41). The first step in selecting the contigs belonging to the genome of *Thermotoga maritima* strain A7A was to assign each of the contigs in the metagenomic assembly to a family by comparing the 25-bp *k*-mers from each contig to all the unique *k*-mers found in ~4,000 microbial genome sequences and draft genome sequences from GenBank. Contigs were also binned using characteristic trinucleotide frequency signatures (2) and covariance principal components analysis (PCA) plots. Where these two methods disagreed, BLAST-based (3) binning was undertaken to confirm the identities of the remaining contigs.

In total, 116 contigs (≥1,034 bp) were assigned to *T. maritima* A7A; the contig lengths ranged from 1,034 to 116,114 bp, with mean, median, and N_50_ lengths of 15,194, 8,670, and 27,335 bp, respectively. In total, the contigs comprise 1,762,576 bp, with an overall G+C content of 45.7%. The closest sequenced relative of *T. maritima* A7A is *T. maritima* strain RQ2 (4), and the two strains share a 16S rRNA gene identity of 100% (over 980 bp). The contigs for the draft genome sequence were annotated using the Integrated Microbial Genomes Expert Review (IMG ER) pipeline (5), which identified 1,987 coding genes. Although *T. maritima* A7A is an anaerobe, like other *T. maritima* strains, it possesses an NAD(P)H oxidoreductase, rubredoxin, the flavodiiron protein, and neelaredoxin, encoded by genes laterally acquired from the archaeal order *Thermococcales* (6), which confer tolerance to low levels of oxygen.

Previous work has found that *Thermotoga* species boast a large number of carbohydrate-active enzymes relative to their genome size (7) and that species within the genus differ in their possession of these enzymes (8). The genome appears to contain genes for the utilization of various disaccharides: sucrose, cellobiose, and maltose, along with the monosaccharides fructose, glucose, and xylose. Further analysis of carbohydrate-active enzymes using the dbCAN web service (9) indicates that in terms of carbohydrate usage, *T. maritima* A7A is most similar to *T. maritima* RQ2, although *T. maritima* A7A appears to lack the fructose phosphotransferase system (PTS) that is present in RQ2. Compared with the other *Thermotoga* species, *T. maritima* A7A appears to possess two carbohydrate-active enzymes not previously described in the genus, an esterase from family CE12 and a glycosyltransferase from family GT27.

Along with *T. maritima* A7A, the microbial community at the A7A well is dominated by species from the genera *Thermoanaerobacter* (10) and *Desulfonauticus*. Examinations of the putative interactions among these organisms in this environment are ongoing.

### Nucleotide sequence accession numbers.

This whole-genome shotgun project has been deposited at DDBJ/EMBL/GenBank under the accession no. AUNF00000000. The version described in this paper is version AUNF01000000.
